# Comparison of European regionwide and Japanese nationwide surveys on gynecologic robotic surgery

**DOI:** 10.1007/s11701-026-03825-4

**Published:** 2026-09-17

**Authors:** Takashi Takeda, Sergi Fernandez-Gonzalez, Misa Hayasaka, Mihoko Dofutsu, Takuma Yoshimura, Ayako Taima, Hiroaki Komatsu, Christina Uwins, Charlotte Chollet, Dina El-Hamamsy, Daniel Galvin, Manou Manpreet Kaur, Eleni Karatrasoglou, Alberto Munoz Solano, Anumithra Amirthanayagam, Hiroaki Kobayashi

**Affiliations:** 1 Department of Women’s and Reproductive Health, University of Oxford, John Radcliffe Hospital, Headington, Oxford, UK; 2 Department of Obstetrics and Gynecology, Keio University School of Medicine, Tokyo, Japan; 3 Department of Gynaecology, University Hospital of Bellvitge (IDIBELL), Hospitalet de Llobregat, Spain; 4 Department of Obstetrics and Gynecology, Asahikawa Medical University, Asahikawa, Japan; 5 Department of Obstetrics and Gynecology, Yokohama City University Medical Center, Yokohama, Japan; 6 Department of Obstetrics and Gynecology, Hirosaki University School of Medicine, Hirosaki, Japan; 7 Department of Obstetrics and Gynecology, Faculty of Medicine, Tottori University, Yonago, Japan; 8 University Hospital Southampton NHS Foundation Trust, Southampton, UK; 9 Department of Gynaecology, Centre Hospitalier Universitaire de Toulouse, Toulouse, France; 10 Department of Obstetrics and Gynaecology, Royal London Hospital, London, UK; 11 Department of Urogynaecology, Cork University Maternity Hospital, Cork, Ireland; 12 Department of Obstetrics and Gynaecology, Chelsea and Westminster Hospital, London, UK; 13 Euroclinic Group of Hospitals Athens, Alexandra University Hospital, Athens, Greece; 14 Gynecology and Obstetrics Service, Hospital Universitario Marqués de Valdecilla, Santander, Spain; 15 Leicester Cancer Research Centre, University of Leicester, Leicester, UK; 16 Department of Obstetrics and Gynecology, Faculty of Medicine, Kagoshima University, Kagoshima, Japan; 17 Robotic Surgery Center, Department of Obstetrics and Gynecology, Munakata Suikokai General Hospital, Fukuoka, Japan; 18 Department of Obstetrics and Gynecology, Keio University School of Medicine, 35 Shinanomachi, Shinjuku-ku, Tokyo, 160-8682 Japan

**Keywords:** Robotic surgical procedures, Education, Gynecology, Surveys and questionnaires, Internship and residency

## Abstract

Gynecologic robotic surgery is increasingly adopted worldwide, and effective surgical education is essential for expanding training opportunities among early-career surgeons. Previously published surveys conducted by the Young European Advocates of Robotic Surgery (YEARS) and the Japan Society of Gynecologic Robotic Surgery (JSGRS) evaluated robotic surgery in Europe and Japan. This comparative analysis explored opportunities for international educational collaboration. This study was a secondary comparative analysis using two previously published survey datasets. The YEARS survey targeted early-career European gynecologists (< 4 years post-fellowship), whereas the JSGRS survey included Japanese obstetricians and gynecologists. Respondents within 12 years after graduation were selected from the JSGRS cohort. A total of 293 respondents were analyzed, including 81 respondents from 21 countries in the YEARS and 212 respondents from the JSGRS survey. The availability of robotic systems was comparable (77.8%vs 74.1% for YEARS and JSGRS, respectively), and interest in robotic surgery was also high in both groups (81.0% vs. 70.3%). Respondents in the YEARS survey reported substantially greater hands-on experience. While 66.7% performed robotic surgery weekly in the YEARS survey, only 3.8% of JSGRS respondents had experience with more than 51 robotic procedures annually. This difference was particularly evident among residents (26.7% vs. 0%). This exploratory comparative analysis suggests that Japanese respondents showed similar interest in robotic surgery to the YEARS cohort but had fewer opportunities for surgical experience. Future studies using standardized multinational surveys are warranted to better characterize regional differences and support the development of robotic surgery education.

**Supplementary Information:**

The online version contains supplementary material available at https://doi.org/10.1007/s11701-026-03825-4.

## Introduction

Robotic surgery has become increasingly widespread in gynecology over the past two decades, driven by technological advancements and expanding clinical applications. Compared with conventional laparoscopy, robotic surgery offers several advantages, including improved ergonomics, enhanced visualization, and a potentially shorter learning curve. The global number of robotic surgical systems has rapidly increased, particularly in North America, Europe, and Asia, reflecting growing adoption of robotic surgery in gynecologic practice.

In Japan, the first robotic hysterectomy was performed in 2009. Although national insurance coverage for robotic hysterectomy in benign disease and endometrial cancer was introduced in 2018, the indications remain limited compared with those in many other countries. Consequently, opportunities for robotic surgical training, particularly among early-career gynecologists, have developed differently from those in Europe [1]. Historically, Europe, especially France, was famous for performing hysterectomy by vaginal and laparoscopic routes as a minimally invasive surgery, which was mainly laparotomy in the US previously. This trend drastically changed to a minimal invasive route after the appearance of the Da Vinci system in the US [2]. The US is the leading country for robotic surgery, although Europe is the second-largest area where robotic surgery is performed. This is also supported by the analysis of the number of academic manuscripts in the gynecologic robotic field [3, 4]. Even though the healthcare system differs in each European country, they attempted to gather evidence of robotic surgery from their countries’ data [5–7].

Previous studies have demonstrated that robotic surgery offers favorable perioperative outcomes [6–9], and a shorter learning curve than conventional laparoscopy [10–13]. Even though there is an advantage to robotic surgery, there is an obstacle for the younger generation to learn and start their robotic surgery in Japan. This is because the delayed insurance coverage has limited the robotic procedure to only experienced, consultant-level gynecologists. To facilitate the widespread of robotic surgery in gynecology, education system in robotic surgical field plays an important role. The Japan Society of Gynecologic Robotic Surgery (JSGRS), which is established in 2018, promotes the safe expansion of robotic surgery through a proctoring system in which certified proctors by the society provide surgical guidance during the introduction of robotic systems at institutions nationwide. And also, the Japan Society of Gynecologic and Obstetric Endoscopy and Minimally Invasive Therapy (JSGOE) established the certification system for gynecological robotic surgical [14]. Meanwhile, the Society of European Robotic Gynaecological Surgery (SERGS), which is established in 2009 has managed European multicentric collaborative tasks to strengthen the scientific weight of their publications in benign and oncological procedures. SERGS put emphasis on education with the policy that the scientific society has to provide a standardized robotic training with the era of increasing robotic platform. They outlined a Pilot Curriculum for standardized education of robotic use in gynecological surgery in 2015 and now they have well-organized education and fellowship programs with lots of webinars and video portals [15]. Young European Advocates of Robotic Surgery (YEARS), young members of SERGS, played an important role in the curriculum, for mentoring and support.

The YEARS group and the young members of the JSGRS independently conducted surveys to assess attitudes toward gynecologic robotic surgery during a similar time period. As a collaborative initiative between these two groups, we performed a secondary comparative analysis of the two previously published surveys to explore similarities and differences in robotic surgical training and education and to identify opportunities for future international collaboration.

## Methods

### Survey datasets

The YEARS group developed a 28-item questionnaire mainly regarding gynecologic surgical training and the surgical activity of both the respondent’s department and the individual surgeon [16]. The survey targeted early-career gynecologic surgeons, including trainee/resident enrolled in a national obstetrics and gynecology training program, gynecology clinical fellow, and recent consultant (within the past 3 years) of a fellowship or subspecialty training program. The survey was hosted on SurveyMonkey and distributed through the social media accounts of YEARS and SERGS from December 2022 to September 2023. In parallel, young members of JSGRS developed the 26-question survey asking about demographic information, current state of robotic surgery implementation, barriers to adoption and skill acquisition, and education in robotic surgery [17]. The survey was distributed to all members of the Japan Society of Obstetrics and Gynecology (JSOG) and collected through Google Forms from September to November 2023. The original questionnaires can be found in Supplement Table 1 (Table.S1).

**Table 1:** Similar questionnaires between the YEARS and JSGRS surveys [16, 17]

Topic	YEARS	JSGRS
Gender	What is your gender?	What is your gender?
Area of Interest / Subspecialty	What is your Gynecological subspecialty or area of interest?	What is your subspecialty?
Facility	Where do you work?	Which type of facility do you work?
Level of experience	What is your level of experience within your current role?	How many years after graduated?
Availability of Robotic Devices	Is there a surgical robotic device at your workplace?	Installation status of robotic surgical systems at the facility
Departmental Robotic Surgery Activity	How often does your department perform robotic surgery?	Annual number of robotic surgeries at the facility
Experience as a Robotic Surgeon	Do you operate as a main surgeon by robotic approach?	Robotic experience as a primary surgeon
Willingness to Perform Robotic Surgery	Would you like to operate more often by a robotic approach?	Do you personally want to be involved in gynecologic robotic surgery?
Learning Opportunities	What learning tools do you access most frequently about robotics (more than one allowed)?	Do you think online education is effective for acquiring robotic surgery skills?

### Selection of comparable cohorts and survey items

To facilitate comparison between the two independently designed surveys with different questionnaires and populations, we selected a subset of respondents for the JSGRS survey to make it more similar to the population of the YEARS survey. In particular, we excluded responses from obstetricians and reproductive doctors, and we included only ≤ 12 years of experience for the JSGRS survey based on the typical postgraduate training pathway in Japan of 2-year general residency, 3-year obstetrics and gynecology training, and an additional 3-year fellowship for gynecologic oncology and minimally invasive surgery, to approximate ≤ 4 years post-fellowship for the YEARS survey. Comparable survey items were selected for comparison analysis, which can be found in Table 1.

### Statistical analysis

For comparisons between the two surveys, Fisher’s exact test was used to compare categorical variables. Statistical significance was set at *p* < 0.05 (*p < 0.05, **p < 0.01, ***p < 0.001). Odds ratios (ORs) with 95% confidence intervals (CIs) were calculated to estimate effect sizes for the principal 2 × 2 comparisons. Variables with more than two categories or conceptually different response options were not subjected to formal statistical comparison and are presented descriptively (NA). Because this was an exploratory secondary analysis comparing two independently designed surveys, no formal adjustment for multiple comparisons was performed. All data manipulations, analyses, and figure construction were conducted using GraphPad Prism version 10.4.1 (GraphPad Software, Boston, MA, USA).

## Results

81 respondents from 55 centers across 21 European countries were included in the YEARS survey, and detailed information on the original survey has been reported previously [16]. Although YEARS and SERGS had approximately 65 and 421 members at the time of the survey, the questionnaire was distributed through their social media platforms and was accessible beyond their membership. Consequently, the number of individuals who received the survey invitation was unknown, and the response rate could not be calculated. In contrast, the original JSGRS survey included 1,443 responses from obstetricians and gynecologists among 17,444 JSOG members, corresponding to a response rate of 8.3% [17]. After restricting the JSGRS dataset to respondents within 12 years after graduation and excluding those primarily practicing obstetrics or reproductive medicine, 212 respondents were included in the present analysis.

**Fig. 1:**
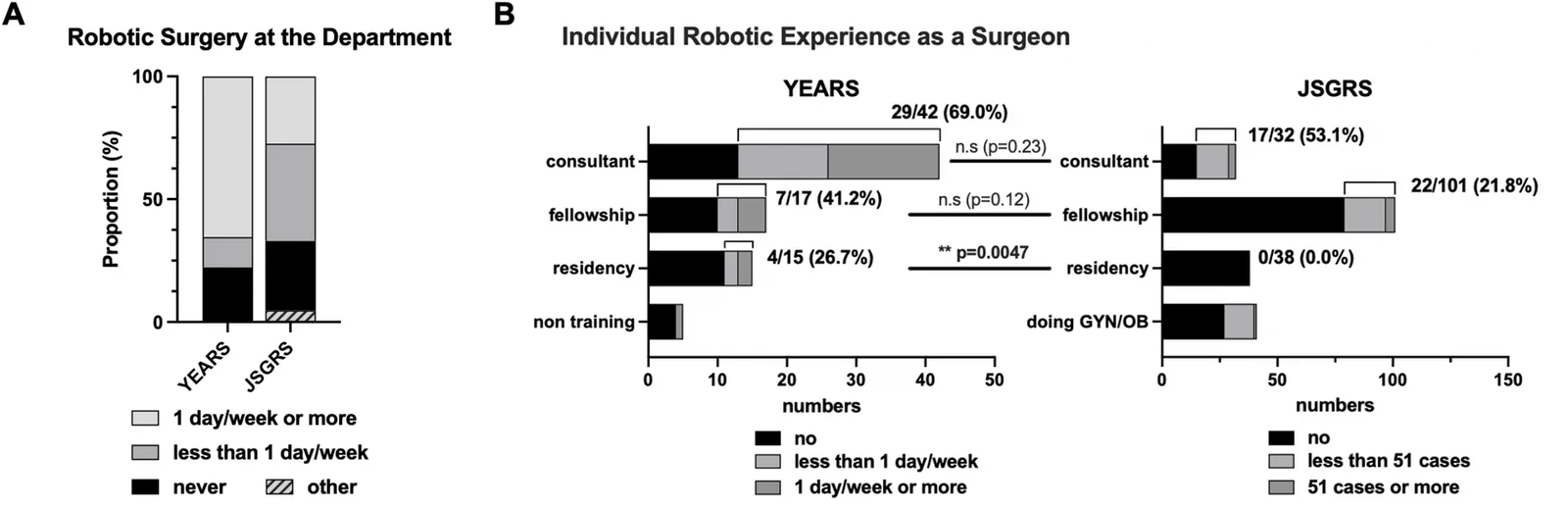
Comparison of robotic surgical volume in European and Japanese surveys. **A**: Comparison of institutional robotic surgery activity between the YEARS and JSGRS surveys. Robotic system availability was similar between the two cohorts. However, higher institutional activity was observed in the YEARS survey, with more centers performing robotic surgery at least once per week, while the proportion of institutions not performing robotic surgery was comparable. **B**: Comparison of individual robotic surgical experience stratified by career stage in the YEARS and JSGRS surveys. The proportion of robotic surgical experience is similar in consultants, fellows level, while the difference was most pronounced at the resident level (*p* = 0.0047)

The baseline characteristics of the two survey cohorts are summarized in Table 2. The gender distribution was comparable between the two surveys, with females accounting for 44/81 (54.3%) in the YEARS survey and 119/212 (56.1%) in the JSGRS survey (*P* = 0.69, OR 0.90 [95%CI 0.54–1.53]). Gynecologic oncology was the predominant subspecialty or interest in both surveys, representing 57/81 (70.4%) in the YEARS survey and 116/212 (54.7%) in the JSGRS survey. Most respondents in the YEARS survey were affiliated with tertiary university hospitals, 47/63 (74.6%). In contrast, in the JSGRS survey, a substantial proportion were affiliated with tertiary non-university centers 110/212 (51.9%), exceeding those in university hospitals 91/ 212 (42.9%), which was statistically significant. In terms of experience, a higher proportion of respondents in the YEARS survey were early-career consultants 43/81 (53.1%), while in the JSGRS survey, the largest group consisted of fellows 101/212 (47.6%), which was significantly different. The availability of robotic surgical systems was similar between the two surveys, reported by 63/81 (77.8%) in the YEARS survey and 157/212 (74.1%) in the JSGRS survey (*P* = 0.55, OR 1.23 [95%CI 0.68–2.19]). With respect to institutional robotic surgery activity, 32/81 (39.5%) in the YEARS survey reported performing robotic surgery more than once per week, whereas in the JSGRS survey, the most common category was 11–50 cases per year 72/212 (34.0%), and performing robotic surgery once per week or more which corresponds to an annual volume of ≥ 51 cases, was higher in the YEARS than JSGRS survey (53/81 [65.4%] vs. 58/202 [28.7%], *p* < 0.001, OR 4.70 [95%CI 2.66–8.04]). The proportion of the facility that never performed robotic surgery was similar between the two surveys (18/81 [22.2%] vs. 60/202 [29.7%], *p* = 0.24, OR 0.68 [95%CI 0.38–1.25]). These findings were consistent with the reported availability of robotic systems at participating institutions (Fig. 1A). For individual surgical experience, a marked difference was observed between the two surveys. In the YEARS survey, 54/81 (66.7%) of respondents reported operating as a main surgeon at least once per week. In contrast, only 8/212 (3.8%) JSGRS respondents reported experience with more than 51 robotic procedures, which is equivalent to once per week for a year. 159/212 (75.0%) reported no experience as a primary robotic surgeon in Japan. To account for differences in career stage between the two cohorts, surgical experience was further analyzed according to training level. In the YEARS cohort, 69.0% of consultants (29/42), 41.2% of fellows (7/17), and 26.7% of residents (4/15) reported performing robotic surgery. In contrast, in the JSGRS cohort, 53.1% of consultants (17/32) and 21.8% of fellows (22/101) had experience as a primary robotic surgeon, which was not significantly different from YEARS, whereas no residents (0/38) reached this level (*p* = 0.0047) (Fig. 1B).

**Table 2:** Characteristics of respondents and their comparison [16, 17]

Characteristics of respondents and their comparison							
YEARS (*n* = 81)	*n*	(%)	JSGRS (*n* = 212)	*n*	(%)	*p*-value	OR (95%CI)
**What is your gender?**			**What is your gender?**				
Female	44	54.3%	Female	119	56.1%	n.s	0.90
Male	37	45.7%	Male	90	42.5%		(0.54–1.53)
			Prefer not to say	3	1.4%		
**What is your Gynecological subspecialty or area of interest?**			**What is your subspecialty?**				
Gynecology Oncology	57	70.4%	Gynecology Oncology	116	54.7%	n.s	
Benign gynecology	18	22.2%	General gynecology	62	29.2%		
Urogynecology	6	7.4%	Women’s Healthcare	27	12.7%		
			Other	7	3.3%		
**Where do you work? (Among 63 responders from where ** **robotic device available)**			**Which type of facility do you work?**				
Tertiary University Hospital	47	74.6%	Tertiary University Hospital	91	42.9%	***	
Tertiary No-University Center	9	14.2%	Tertiary No-University Center	110	51.9%		
Private Hospital	7	11.1%	Private Hospital	7	3.3%		
			Others	4	1.9%		
**What is your level of experience within your current role?**			**How many years after graduated? (< 12Y)**				
Currently, doing training program in gyn/obs	16	19.8%	Residency (-5Y)	38	17.9%	***	
Currently, 1/2 half of fellowship	12	14.8%	Fellowship (5Y- without subspeciality)	101	47.6%		
Currently, 2/2 half of fellowship	5	6.2%	Consultant (5Y- with certification of subspeciallty)	32	15.1%		
Working as a consultant < 4 years	43	53.1%	Other (5Y-, doing general)	41	19.3%		
Others	5	6.2%					
**Is there a surgical robotic device at your workplace?**			**Installation status of robotic surgical systems at the facility**				
Yes	63	77.8%	Yes	157	74.1%	n.s	1.23
No	18	22.2%	No	55	25.9%		(0.68–2.19)
**How often does your department perform robotic surgery?**			**Annual number of robotic surgeries at the facility (Case/ Year)**				
> 1 day per week	32	39.5%	151-	5	2.4%	NA	
1 day per week	21	25.9%	101–150	12	5.7%		
< 1 day per week	10	12.3%	51–100	41	19.3%		
Never	18	22.2%	11–50	72	34.0%		
			1–10	12	5.7%		
			Never	60	28.3%		
			Not well known	10	4.7%		
**Departmental robotic surgery (frequent or not)**							
Once per week or more	53	65.4%	Annual volume of ≥ 51 cases	58	28.7%	***	4.70
Less	28	34.6%	Less	144	71.3%		(2.66–8.04)
**Departmental robotic surgery (Never or not)**							
Never	18	22.2%	Never	60	29.7%	n.s	0.68
Not	63	77.8%	Not	142	70.3%		(0.38–1.25)
**Do you operate as a main surgeon by robotic approach?**			**Robotic experience as a primary surgeon (Case so far)**				
< 1 day per week	39	48.1%	101–150	2	0.9%	NA	
1 day per week	15	18.5%	51–100	6	2.8%		
> 1 day per week	8	9.9%	11–50	28	13.2%		
No device	18	22.2%	1–10	17	8.0%		
NA	1	1.2%	Never	159	75.0%		
**Would you like to operate more often by a robotic approach? (Among 63 responders)**			**Do you personally want to be involved in gynecologic robotic surgery?**				
No, I am happy with my frequency	11	17.5%	Strongly agree	67	31.6%	NA	
I would like to operate more often as a main surgeon	35	55.6%	Slightly agree	82	38.7%		
I would like to initiate surgeries as a main surgeon	16	25.4%	Not sure	6	2.8%		
NA	1	1.5%	Slightly disagree	38	17.9%		
			Strongly disagree	19	9.0%		
**What learning tools do you access most frequently about robotics (more than one allowed)?**			**Do you think online education is effective for acquiring robotic surgery skills?**				
On-site courses	34	42.0%	Strongly agree	53	25.0%	NA	
Webinars	40	49.4%	Slightly agree	116	54.7%		
Society websites	27	33.3%	Not sure	13	6.1%		
Social media	12	14.8%	Slightly disagree	27	12.7%		
			Strongly disagree	3	1.4%		

NA: not applicable, n.s: not significant, OR: Odds ratio, CI: confidence intervals, ***p < 0.001

Despite these differences, interest in robotic surgery was high in both groups. In the YEARS survey, 51/63 (81.0%) expressed a desire to perform more robotic procedures as a main surgeon or to initiate such surgeries, while in the JSGRS survey, 149/212 (70.3%) either strongly or slightly agreed that they wished to be involved in robotic surgery. Regarding educational aspects, both surveys demonstrated positive engagement with online educational resources. In the YEARS survey, webinars were accessed by 40/63 (49.4%) of respondents, while in the JSGRS survey, 169/212 (79.7%) of respondents considered online education to be effective for acquiring robotic surgery skills.

## Discussion

In this comparative analysis of early-career gynecologic surgeons from Europe and Japan, several key findings emerged. While institutional access to robotic surgery was comparable between the two regions, higher institutional activity and earlier acquisition of robotic surgical experience were observed in the YEARS cohort, suggesting potential differences in training structures and opportunities. These findings may reflect differences in training systems and clinical environments rather than differences in access to robotic platforms alone. Early opportunities for exposure to robotic surgery may play a critical role in determining surgical experience among early-career surgeons. Nevertheless, interest in robotic surgery and engagement with educational resources remained consistently high across both cohorts.

Early exposure to robotic surgery is increasingly recognized as essential for efficient skill acquisition and the safe implementation of robotic procedures. Structured training programs incorporating simulation, stepwise progression, and objective assessment have been shown to improve surgical performance and shorten the learning curve, particularly in minimally invasive surgery [18–20]. In Europe, the SERGS has pioneered the standardization of robotic education. The SERGS curriculum emphasizes a stepwise, competency-based approach, moving from simulation and modular dry-lab training to supervised clinical practice [15, 21–23]. Such structured educational pathways may partly explain the greater hands-on experience observed among respondents in the YEARS cohort.

The global expansion of robotic surgery has been accompanied by a rapid increase in the number of robotic surgical systems worldwide, particularly in North America, Europe, and Asia. Significant growth was also observed in YEARS survey (from 264 to 1,199) and Asia (from 84 to 1,050) during the same period [24–26]. Furthermore, numerous global and domestic companies have developed original robotic platforms. However, despite similar availability of robotic systems observed in this study, substantial differences in institutional activity and individual surgical experience remained between the two regions. This discrepancy suggests that system availability alone is insufficient to ensure adequate surgical exposure. Differences in institutional structure, including the distribution of university and non-university centers, may also contribute to variations in training opportunities and surgical exposure. These findings highlight the necessity of not only structured training frameworks but also clinical environments that actively provide these opportunities to trainees.

In contrast, robotic surgical training systems in Japan are still evolving. Although certification systems for robotic surgery have been introduced alongside established endoscopic skill certification frameworks, society-based educational programs remain less standardized than those reported in Europe [14]. Furthermore, limitations in case distribution and restricted indications under the national healthcare system may further reduce opportunities for early-career surgeons to gain hands-on experience. On a broader scale, the Asian Society of Gynecologic Robotic Surgery (ASGRS) has begun to address these gaps by establishing regional robotic education systems aimed at harmonizing training standards across Asia, reflecting an increasing recognition of the importance of formalized robotic training systems beyond Europe [27]. Such a framework, integrating simulation, objective assessment, and regional collaboration, will be vital to nurturing the next generation of robotic surgeons and ensuring the continued evolution of gynecologic surgery.

Several limitations of this study should be acknowledged. First, the analysis was based on a comparison of two independently conducted surveys with different designs, target populations, and question formats. In addition, the recruitment strategies also differed substantially between the two surveys. The YEARS survey was disseminated through the social media accounts of YEARS and SERGS, whereas the JSGRS survey was distributed through the mailing list of the society’s members. Because the total number of individuals who received or viewed the YEARS survey invitation was unknown, the response rate could not be calculated, limiting the assessment of the representativeness of the YEARS survey respondents. Furthermore, these different recruitment strategies may have introduced different forms of selection bias. To identify a cohort comparable to surgeons with ≤ 4 years after fellowship in Europe, we selected the most comparable cohort within the Japanese dataset based on the structure of postgraduate training in Japan. Physicians complete a 2-year general residency before entering an obstetrics and gynecology training program, which typically lasts 3 years. After board certification in obstetrics and gynecology, subspecialty training in both gynecologic oncology and minimally invasive surgery generally requires an additional 3 years, and these fellowships are often undertaken concurrently. Consequently, completion of fellowship training usually requires approximately 8 years after graduation. Based on this training pathway, we selected surgeons within 12 years after graduation to approximate the YEARS cohort. This selection is supported by the finding that the proportion of consultant surgeons with robotic surgery experience was not significantly different between the two cohorts. Although we selected comparable items and curated the data to align the study populations, the comparison remains indirect and may not fully capture regional differences in training and clinical practice. Finally, because this study was based on secondary analyses of previously published survey datasets, information regarding the handling of incomplete questionnaires and duplicate responses was limited to that reported in the original studies and could not be independently verified. Second, the variables assessed in each survey were not completely identical, particularly regarding the measurement of surgical experience and institutional activity, which may have influenced the interpretation of the results. Because of these limitations, future studies using standardized questionnaires are warranted to enable more direct regional comparisons. We are currently planning further collaborative surveys across Asia, eventually across broader international regions, using a standardized and unified questionnaire. Such efforts will allow for a more accurate evaluation of regional differences and contribute to the development of optimized training systems in robotic gynecologic surgery, ultimately bridging the gap between surgical interest and practical experience while ensuring equitable access to robotic surgical training across regions.

## Conclusion

In conclusion, this exploratory comparative analysis suggests that early-career gynecologic surgeons in Japan share a similarly high level of interest in robotic surgery as YEARS survey showed. However, despite comparable access to robotic platforms, Japanese surgeons experience fewer opportunities for hands-on training and surgical practice. Although the comparison was based on two independently designed surveys and should therefore be interpreted with caution, these findings may suggest differences in training opportunities between the two regions. Further multinational surveys using standardized questionnaires, particularly including a broader range of countries, are warranted to better understand regional differences and identify underlying challenges. Such efforts may help inform the development of standardized and effective training frameworks, ultimately promoting the wider dissemination of robotic surgery in gynecology.

## Supplementary Information

Below is the link to the electronic supplementary material.

Supplementary Material 1

## Data Availability

No datasets were generated or analysed during the current study.
